# Nontransformation methods for studying signaling pathways and genes involved in *Brassica rapa* pollen–stigma interactions

**DOI:** 10.1093/plphys/kiae445

**Published:** 2024-08-30

**Authors:** Lili Zhang, Xiaoshuang Cui, Lin Yang, Abdul Raziq, Shiya Hao, Weiqing Zeng, Jiabao Huang, Yunyun Cao, Qiaohong Duan

**Affiliations:** College of Horticulture Science and Engineering, Shandong Agricultural University, Tai’an 271018, China; College of Horticulture and Landscape Architecture, Yangzhou University, Yangzhou 225009, China; College of Horticulture Science and Engineering, Shandong Agricultural University, Tai’an 271018, China; College of Horticulture Science and Engineering, Shandong Agricultural University, Tai’an 271018, China; College of Horticulture Science and Engineering, Shandong Agricultural University, Tai’an 271018, China; Directorate of Vegetable Seed Production, Agriculture Research Institute, Village Aid Sariab, Quetta, 87300 Balochistan, Pakistan; College of Horticulture Science and Engineering, Shandong Agricultural University, Tai’an 271018, China; Health and Biosciences, International Flavors and Fragrances, Wilmington, DE 19803, USA; College of Horticulture Science and Engineering, Shandong Agricultural University, Tai’an 271018, China; Institute of Vegetable Science, Zhejiang University, Hangzhou 310058, China; College of Horticulture Science and Engineering, Shandong Agricultural University, Tai’an 271018, China; College of Horticulture Science and Engineering, Shandong Agricultural University, Tai’an 271018, China

## Abstract

Self-incompatibility (SI) is a mechanism in plants that prevents self-fertilization and promotes outcrossing. SI is also widely utilized in the breeding of Brassicaceae crops. Understanding the regulatory mechanisms of SI is essential but has been greatly restrained in most Brassicaceae crops due to inefficient transformation. In this study, we developed methods for examining signaling pathways and genes of pollen–stigma interactions in Brassicaceae crops lacking an efficient genetic transformation system. We pretreated excised stigmas of *Brassica rapa* (*B. rapa* L. ssp. *Pekinensis*) in vitro with chemicals to modify signaling pathways or with phosphorothioate antisense oligodeoxyribonucleotides (AS-ODNs) to modify the expression of the corresponding genes involved in pollen–stigma interactions. Using this method, we first determined the involvement of reactive oxygen species (ROS) in SI with the understanding that the NADPH oxidase inhibitor diphenyleneiodonium chloride, which inhibits ROS production, eliminated the SI of *B. rapa*. We further identified the key gene for ROS production in SI and used AS-ODNs targeting *BrRBOHF* (*B. rapa RESPIRATORY-BURST OXIDASE HOMOLOGF*), which encodes one of the NADPH oxidases, to effectively suppress its expression, reduce stigmatic ROS, and promote the growth of self-pollen in *B. rapa* stigmas. Moreover, pistils treated in planta with the ROS scavenger sodium salicylate disrupted SI and resulted in enlarged ovules with inbred embryos 12 d after pollination. This method will enable the functional study of signaling pathways and genes regulating SI and other pollen–stigma interactions in different Brassicaceae plants.

## Teaser

Treating stigmas with chemical and antisense oligodeoxyribonucleotides enables the study of signaling pathways and genes in pollen–stigma interactions in Brassicaceae crops lacking a transgenic system.

## Introduction

When a flower blooms, the stigma of the pistil has to respond properly to the pollen grains landing on it, allowing the growth of only compatible pollen but rejecting incompatible pollen to prevent the formation of unfavorable progeny (Huang et al. 2023). Many aspects of these pollen–stigma interactions have been studied in recent decades in an attempt to understand how the decisions of compatibility/incompatibility are made, as well as strategies used in the promotion and rejection of pollen (Dresselhaus and Franklin-Tong 2013; Zhong Qu 2019; Goring et al. 2023; Nasrallah 2023), but we still know rather little about some of the events involved. Self-incompatibility (SI) is one of these pollen–stigma interactions that recognizes and suppresses the growth of self-pollen to prevent inbreeding depression (Gibbs 2014; Abhinandan et al. 2022; Goring et al. 2023; Zhang et al. 2024). In the Brassicaceae, the recognition of self-pollen occurs through the interaction between an *S*-locus cysteine-rich protein (SCR) in the pollen coat and the *S*-locus Ser/Thr receptor kinase (SRK) in the cell membrane of the stigma papilla (Shimosato et al. 2007; Ma et al. 2016; Nasrallah 2019; Ohata et al. 2023). The recognition of self-pollen initiates downstream signal cascades that ultimately lead to the rejection of the self-pollen (Jany et al. 2019; Nasrallah 2019).

The identification of genes involved in SI and the mechanistic study of their functions rely on the combination of forward genetic approaches, such as the use of T-DNA insertion (Errampalli et al. 1991) and mutagens (EMS, Kim et al. 2006), and reverse genetic approaches such as genetic transformation to up- or downregulate the expression of corresponding genes (Sankaranarayanan et al. 2015; Scandola and Samuel 2019; Kenney et al. 2020; Zhang et al. 2023). Therefore, research on Brassicaceae SI has predominantly focused on plants like *Brassica napus* L. (Sankaranarayanan et al. 2015; Scandola and Samuel 2019; Abhinandan et al. 2023), which has an efficient genetic transformation system, and *Arabidopsis* (*Arabidopsis thaliana*) expressing cognate SCR of pollen and the SRK of stigma that recapitulates SI (Zhang et al. 2019; Lin et al. 2022; Macgregor et al. 2022; Li et al. 2023). However, research on SI mechanisms in Chinese cabbage and other Brassicaceae crops lacking efficient genetic transformation systems has been greatly restrained.

Transient inhibition of enzyme activities and inhibition of gene expressions are widely applied in various functional studies. In *Arabidopsis*, feeding excised pistils from the cut pedicle with diphenyleneiodonium chloride (DPI), which specifically inhibits the activity of NADPH oxidases in producing reactive oxygen species (ROS) in the ovule, impaired pollen tube rupture, indicating that ROS are required for pollen tube rupture in the synergid cells (Duan et al. 2014). In pear pollen culture, adding increasing concentrations of EGTA to chelate Ca^2+^ progressively reduced the RNA content of pollen tubes, indicating that Ca^2+^ concentrations affect RNA levels in pear pollen tubes (Qu et al. 2012). In *B. rapa*, feeding excised stigmas with GDP, which converts Rac/Rop GTPases into predominantly inactive form, drastically weakened the strength of SI, indicating that Rac/Rop signaling pathways are involved in SI responses in *B. rapa* (Zhang et al. 2021). The above-mentioned method provides researchers with a convenient approach to explore the pathways of pollen–stigma interactions.

Antisense oligodeoxyribonucleotides (AS-ODNs) have recently been successfully applied to plant cells, including pollen tubes (Mizuta and Higashiyama 2014; Cao et al. 2022) and stigmas (Huang et al. 2020; Su et al. 2020). AS-ODNs, which are designed to be complementary to hypervariable regions of mRNA of the target gene, hybridize to its cognate mRNA and induce the inhibition of gene expression. A few bases at both the 5′ and the 3′ end of S-ODN and AS-ODN were phosphorothioate-modified to maintain stability, resistant to a variety of nuclease activities in the cell (Gheibi-Hayat and Jamialahmadi 2021; Wdowikowska and Janicka 2021). In pear pollen culture, incompatible PbrS-RNase disrupted actin cytoskeleton dynamics, but adding AS-ODNs that specifically suppress the expression of *phospholipase D* (*PbrPLDδ1*) is important for accelerating actin depolymerization, indicating that PbrPLDδ1 functions in pear SI by stabilizing the actin cytoskeleton (Chen et al. 2018). In *B. rapa*, adding AS-ODNs that specifically suppress the expression of a negative regulator of ethylene responses, *BrCTR1*, caused programmed cell death in papilla cells and broke down SI, indicating that ethylene negatively mediates SI response (Su et al. 2020). In *Arabidopsis*, AS-ODNs inhibited pollen tube growth–related genes *ANX1*, *ANX2*, *ROP1*, and *CalS5*, resulting in a substantial reduction in *Arabidopsis* pollen tube growth (Mizuta and Higashiyama 2014). AS-ODNs targeting *S*-locus determinants *Pr-p26.1a* and *Pr-p26.1b* in *Papaver rhoeas* significantly increased pollen tube length, emphasizing the critical role of these genes in the SI process (de Graaf et al. 2006). The above-mentioned method allows for the transient suppression of key gene expression using AS-ODNs, providing a powerful tool for validating the functionality of key genes in the pathway of pollen–stigma interactions.

The above-described approaches are suitable for pollen–stigma interactions such as compatibility, SI, and interspecies barriers, because the entire process, from pollen hydration and germination to pollen tube penetration into papilla cells, usually completes within 2 h (Zhang et al. 2021; Huang et al. 2023). This provides us the convenience to study pollen responses in stigmas with transiently altered environments both in vitro and in planta. Here, we report effective approaches, including the pretreatment of Chinese cabbage stigmas with chemicals or AS-ODNs, to investigate pollen–pistil interactions. These approaches will facilitate the study of signaling pathways and key genes regulating pollen–stigma interactions in Chinese cabbage and other Brassicaceae crops.

## Results

### Two feeding methods for stigma treatment

**Figure 1.**
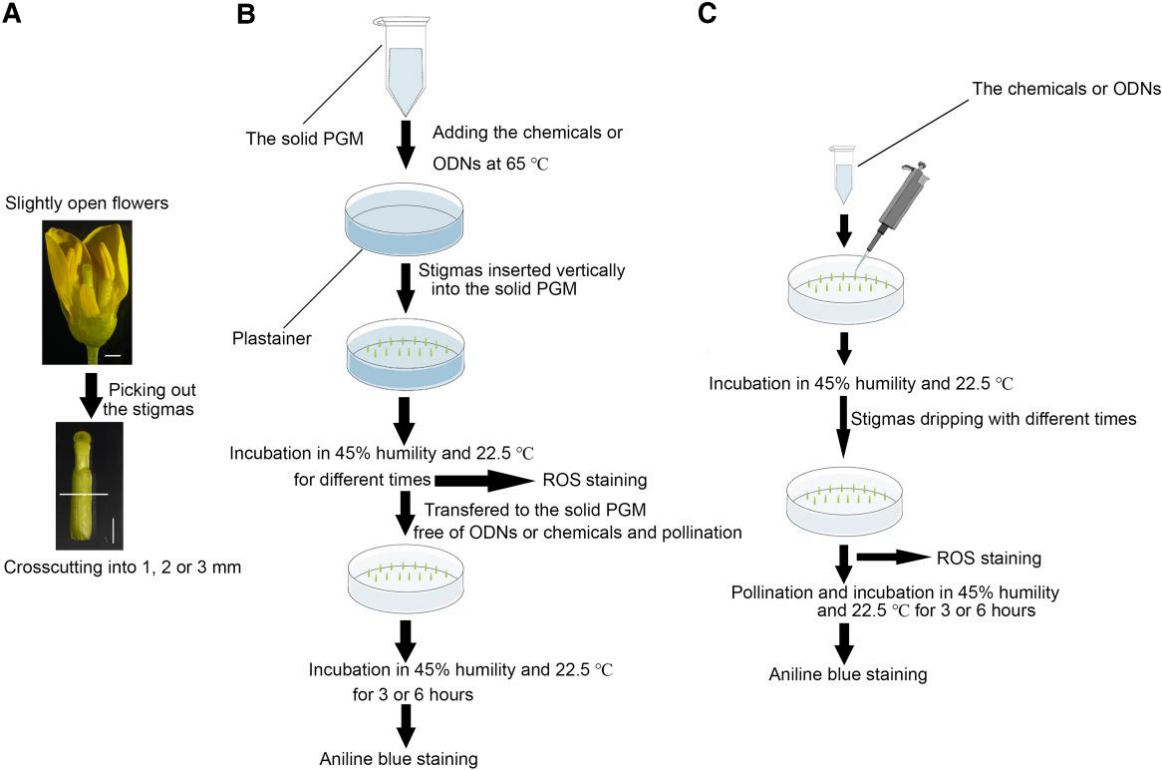
A schematic diagram of stigma treatment methods for pollen–stigma interaction studies. **A)** Flowers and stigmas for bottom-feeding and top-dripping methods. The stigmas were cross-sectioned into the desired length. **B)** Stigmas feeding an assay. The chemicals or AS-ODNs were dissolved in the medium. The stigmas were inserted into the pollen germination media (PGM) and incubated at the indicated humidity and temperature. **C)** A top-dripping assay with the treatment of chemicals or AS-ODNs. Tween 20 at a final concentration of 0.0125% was added to the dripping reagents. Scale bar = 1 mm.

We developed 2 methods, namely, “bottom feeding” and “top dripping,” to study various pollen–stigma interactions in *B. rapa*. With the “bottom-feeding” method, *B. rapa* stigmas from flowers at stages analogous to the *A. thaliana* Stage 12 to Stage 13 (Smyth et al. 1990; Fig. 1A). Chemicals, -oligodeoxyribonucleotides (ODNs), or control solutions were added to pollen germination media (PGM) when it was cooled to 65 °C, and the mixed medium was aliquoted in small petri dishes or the caps of 1.5 mL microfuge tubes. The stigmas were then inserted into the cooled medium and placed in a chamber maintained at a constant temperature of 22.5 °C and a humidity of 45% for the designed treatment duration. After the treatment, the stigmas were stained with H_2_DCFDA to detect ROS or transferred to a basic PGM and then pollinated with a layer of self-pollen or cross-pollen. After pollination, the stigmas were kept under the same conditions for a designated time and then stained with aniline blue to visualize pollen tubes (Fig. 1B). In the “top-dripping” method, chemicals and ODNs were prepared at designated concentrations in solvents containing 0.0125% Tween 20 and then dripped dropwise onto the surface of the stigmas that had been inserted into PGM plates (Fig. 1C). After the desired treatment times, stigmas were stained with H_2_DCFDA and aniline blue for ROS and pollen tube detection, similar to the bottom-feeding method (Fig. 1C).

We first measured the vitality of stigmas to ensure that they were suitable for the treatment. Stigmas treated with a mock solution either with the bottom-feeding method or with the top-dripping method maintained typical SI and compatible pollination (CP) phenotypes, similar to that observed in stigmas growing on the plant (Supplementary Fig. S1), showing that mock treatments did not affect the vitality of stigmas. Second, to visually observe the absorption of chemicals or ODNs by the stigmas, we used red food dye to trace the chemical movement in both feeding methods. Cross sections and longitudinal sections of treated stigmas showed that the red food dye was transported through the internal tissues and successfully absorbed by the stigmatic papilla cells in the bottom-feeding method (Supplementary Fig. S2A). On the other hand, in the top-dripping method, the red food dye was observed to be directly absorbed by the stigmatic papilla cells after application (Supplementary Fig. S2B).

### Determine the optimum concentration for the chemical to break down SI

To test the involvement of ROS in the SI responses of the Brassicaceae plants, we needed to determine the optimum concentration for ROS inhibitors in breaking down SI. *B. rapa* stigmas were treated with different concentrations of the NADPH oxidase inhibitor, DPI, using both the bottom-feeding and the top-dripping methods.

**Figure 2.**
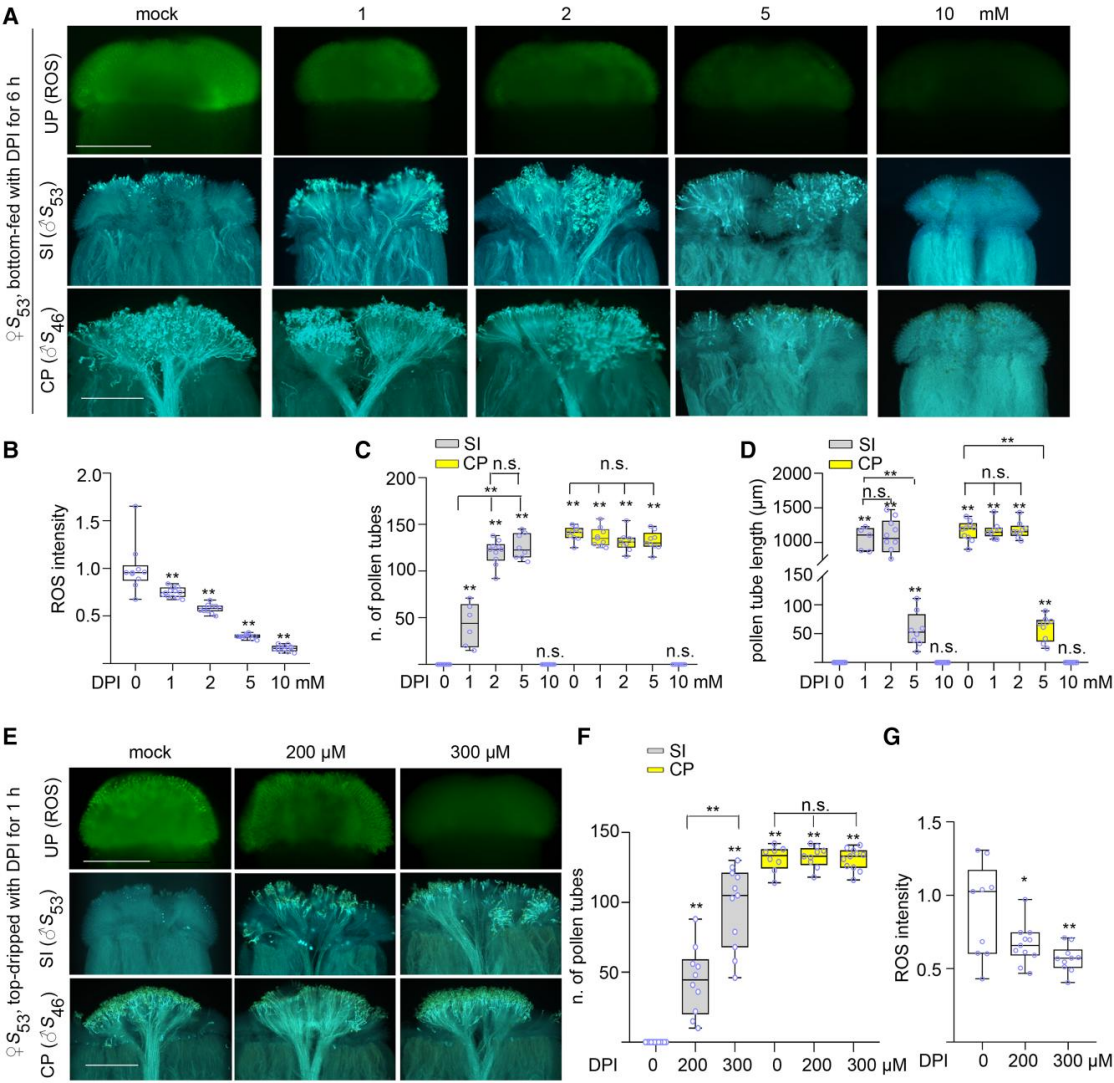
ROS accumulation and the growth of self- or cross-pollen tubes in stigmas treated with different concentrations of DPI with bottom-feeding and top-dripping methods. **A)** ROS accumulation and the growth of self- or cross-pollen tubes in stigmas treated with different concentrations of DPI with the bottom-feeding method. **B)** A quantification of ROS signals observed in **A**. **C)** The numbers of self- or cross-pollen tubes observed in **A**. **D)** The length of self- or cross-pollen tubes observed in **A**. **E)** ROS accumulation and the growth of self- or cross-pollen tubes in stigmas treated with different concentrations of DPI with the top-dripping method. **F)** The numbers of self- or cross-pollen tubes observed in **E**. Boxplots: center line, median; box limits, upper and lower quartiles; whiskers, highest and lowest data points; dots, individual data points. The asterisks in the data indicate a significant difference (2-tailed *t*-test; *P < 0.05; **P < 0.01); n.s. indicates no significant difference (compared with the mock data on the far left). Each experiment was repeated at least 3 times. **G)** A quantification of ROS signals observed in **E**. Scale bar = 500 *µ*m.

In the bottom-feeding method, the stigmas were excised at 3 mm from the papilla cells and treated with 0, 1, 2, 5, and 10 mm DPI for 6 h. Subsequently, the stigmas were stained for ROS or transferred to basic PGM and then pollinated with self- or cross-pollen. The optimal concentration was determined by the reduction of ROS, the numbers and length of self-pollen tubes, together with the comparison with that of the cross-pollen tubes. The reduction of stigmatic ROS by DPI treatment showed a dose-dependent effect ranging from 1 to 10 mm (Fig. 2, A and B). However, the growth of pollen tubes at 6 h after pollination (HAP) was rather complicated. First, an average of 45, 125, and 120 self-pollen tubes penetrated the 1, 2, and 5 mm DPI-treated stigmas, suggesting that DPI broke down SI in a dose-dependent manner from 1 to 2 mm, but reached a plateau at 5 mm DPI (Fig. 2, A and C). Second, the lengths of the self-pollen tubes in 1 and 2 mm DPI-treated stigmas were comparable with that of cross-pollen tubes, but were 20 times longer than those in 5 mm DPI-treated stigmas, suggesting that 5 mm DPI exhibited a severe side effect in inhibiting pollen tube growth (Fig. 2, A and D). Finally, further increasing the DPI concentration to 10 mm led to a complete inhibition of germination and growth of both self- and cross-pollen, as neither self- nor cross-pollens could grow (Fig. 2, A and D). We therefore concluded that 2 mm DPI was the optimum concentration in breaking down SI without causing significant side effects.

In the top-dripping method, 200 and 300 *µ*m DPI were dripped directly onto the surface of the stigmas. Stigmas treated for 1 h were either stained for ROS or pollinated and stained for pollen tubes at 6 HAP. Similar to the bottom-feeding method, 300 *µ*m DPI reduced stigmatic ROS and increased the number of self-pollen tubes to a greater extent than the 200 *µ*m DPI treatment (Fig. 2, E to G). However, neither the 200 *µ*m nor the 300 *µ*m DPI treatment affect the growth of compatible pollen tubes (Fig. 2, E and F). It is worth noting that treatment of 300 *µ*m DPI with the top-dripping method resulted in an average of 100 self-pollen tubes in stigmas, which is comparable with the 2 mm DPI treatment with the bottom-feeding method for 6 h. The reason could be that the chemical can enter the stigma papilla cells more easily in the top-dripping method than in the bottom-feeding method.

Altogether, it is practical to determine the optimum concentration of DPI treatment in both methods based on 2 criteria: selecting the effective concentration for breaking down SI by the number of self-pollen tubes penetrating into the treated stigma; and setting the upper limit of the excess concentration by comparing the number of cross-pollen tubes in the treated stigma to that in the untreated stigma.

### Determine the optimum treatment time for the chemical to break down SI

**Figure 3.**
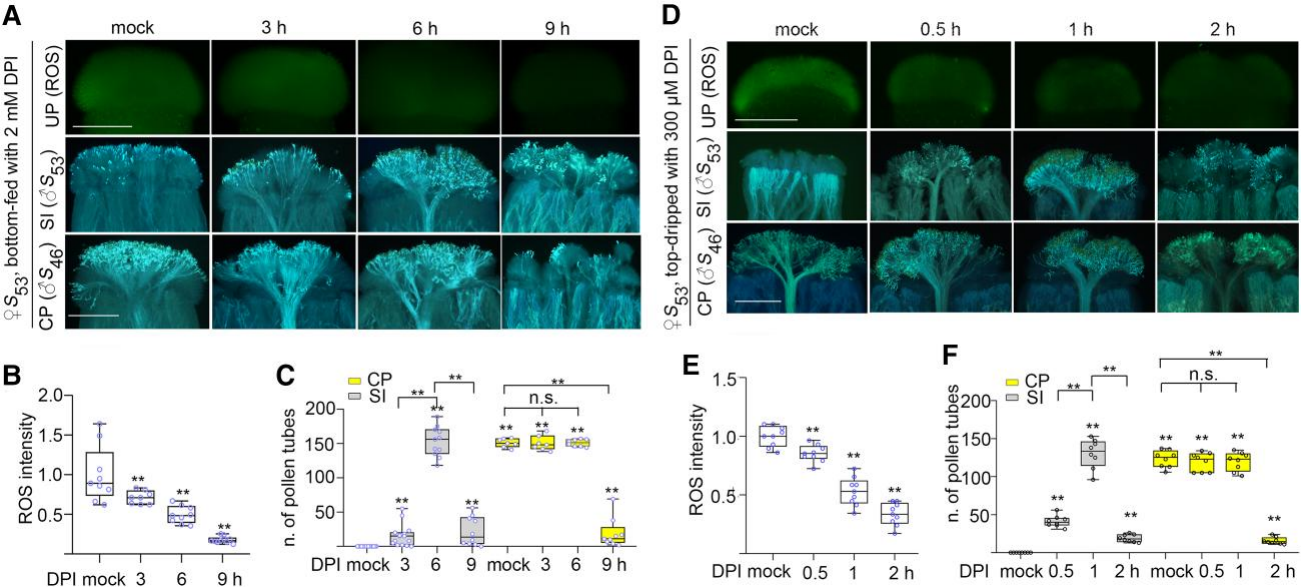
ROS levels and the growth of self- or cross-pollen tubes in stigmas treated with DPI for different durations using bottom-feeding and top-dripping methods. **A)** ROS levels and the growth of self- or cross-pollen tubes in stigmas treated with DPI for different durations using the bottom-feeding method. **B)** A quantification of ROS signals observed in **A**. **C)** The numbers of self- or cross-pollen tubes observed in **A**. **D)** ROS accumulation and the growth of self- or cross-pollen tubes in stigmas treated with different concentrations of DPI using the top-dripping method. **E)** A quantification of ROS signals observed in **D**. **F)** The numbers of self- or cross-pollen tubes observed in **D**. Boxplots: center line, median; box limits, upper and lower quartiles; whiskers, highest and lowest data points; dots, individual data points. The asterisks in the data indicate a significant difference (2-tailed *t*-test; **P < 0.01); n.s. indicates no significant difference (compared with the mock data on the far left). Each experiment was repeated at least 3 times. Scale bar = 500 *µ*m.

To determine the optimum treatment time for DPI to break down SI, we treated 3 mm long *B. rapa* stigmas for different durations with the optimum concentration, i.e. 2 mm in the bottom-feeding method and 300 *µ*m in the top-dripping method. Both methods reduced stigmatic ROS in a dose-dependent manner with increasing treatment time (Fig. 3, A, B, D, and E). Regarding the effect in breaking down SI, 6 h of bottom-feeding treatment with 2 mm DPI and 1 h of top-dripping treatment with 300 *µ*m DPI resulted in the maximum numbers of self-pollen tubes (∼120 to 150), which was significantly more than that of the 3 and 9 h bottom-feeding-treated stigmas, and 0.5 and 2 h top-dripping-treated stigmas (∼20) (Fig. 3, A, C, D, and F). For the upper limit, the numbers of cross-pollen tubes in the stigmas of the 3 and 6 h bottom-feeding treatment (∼150) and stigmas of the 0.5 and 1 h top-dripping treatment (∼125) were comparable with that in the nontreated stigmas, but significantly more than that in the 9 h bottom-feeding-treated stigmas and 2 h top-dripping-treated stigmas (∼20; Fig. 3, A, C, D, and F). Therefore, we determined that the optimum time for 2 mm DPI treatment in the bottom-feeding method was 6 h, and the optimum time for 300 *µ*m DPI treatment in the top-dripping method was 1 h for breaking down SI without inhibiting pollen growth.

### Determine the optimum stigma length for AS-ODN to break down SI

**Figure 4.**
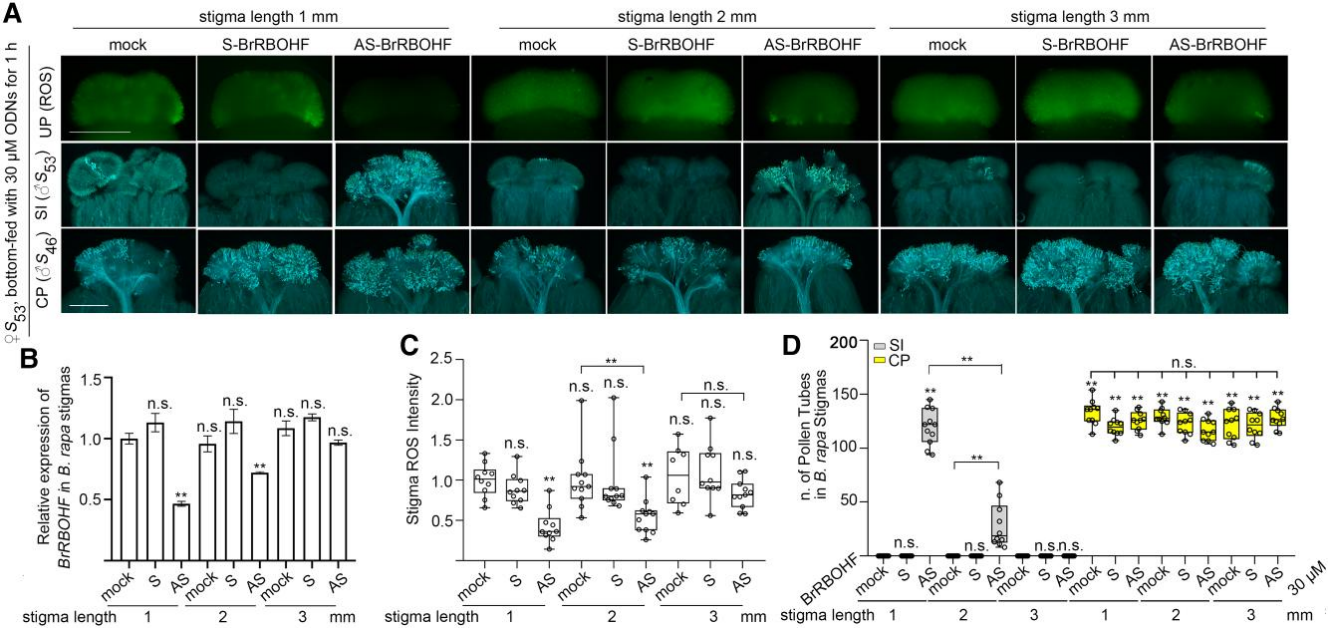
The effect of different stigma lengths on stigmatic ROS accumulation and self- or cross-pollination in *B. rapa* after S- or AS-BrRBOHF treatment with the bottom-feeding method. **A)** ROS accumulation and the growth of self- or cross-pollen tubes in stigmas treated with different lengths of S- or AS-BrRBOHF by the bottom-feeding method. **B)** An RT-qPCR analysis of *BrRBOHF* after treatment with S- or AS-BrRBOHF for different lengths of stigmas. Bar graphs: mean value, ±Sd for gene expression data of *BrRBOHF*. The dots indicate each data point. The asterisks in the data indicate a significant difference (2-tailed *t*-test; *P* < 0.05; **P < 0.01); n.s. indicates no significant difference (compared with the mock data on the far left). Each experiment was repeated at least 3 times. **C)** A quantification of ROS signals observed in **A**. **D)** The numbers of self- or cross-pollen tubes observed in **A**. Boxplots: center line, median; box limits, upper and lower quartiles; whiskers, highest and lowest data points; dots, individual data points. The asterisks in the data indicate a significant difference (2-tailed *t*-test; **P < 0.01); n.s. indicates no significant difference (compared with the mock data on the far left). Each experiment was repeated at least 3 times. Scale bar = 500 *µ*m.

AS-ODN treatment took effect within 1 and 2 h in the bottom-feeding method, but within 40 and 60 min in the top-dripping method, which may be due to the challenges from the endogenous nucleases. We therefore tested the optimum stigma length to reduce the time required to reach the top papilla cells for the bottom-feeding method while still maintaining the original feature of SI and cross-compatibility. In the bottom-feeding method, *B. rapa* stigmas were excised at 1, 2, and 3 mm from the papilla cells and treated for 1 h. In terms of inhibiting BrRBOHF expression and ROS production, 1 and 2 mm long stigmas were both effective, but 3 mm long stigmas were ineffective and did not differ from untreated stigmas (Fig. 4, A to C). Although both 1 and 2 mm stigmas with AS-BrRBOHF treatment successfully broke down SI, the numbers of self-pollen tubes in 1 mm stigmas were 6 times that of the 2 mm stigmas, while the numbers of cross-pollen tubes in treated or untreated stigmas were similar (Fig. 4D). These results suggest that 1 mm is the optimum stigma length for 1 h of 30 *μ*m AS-BrRBOHF bottom-feeding treatment.

### Determine the optimum concentration for AS-ODN to break down SI

Results from DPI treatment in the above-mentioned experiments guided us to further explore key genes encoding NADPH oxidases regulating SI in *B. rapa*. Since the efficient transformation of Chinese cabbage is still difficult to accomplish, we used AS-ODNs to transiently suppress the expression of *BrRBOHs* encoding NADPH oxidases in *B. rapa* stigmas (Zhang et al. 2021; Huang et al. 2023). *BrRBOHF* is the most highly expressed *BrRBOHs* in the stigma, we therefore designed the corresponding S- and AS-ODNs.

**Figure 5.**
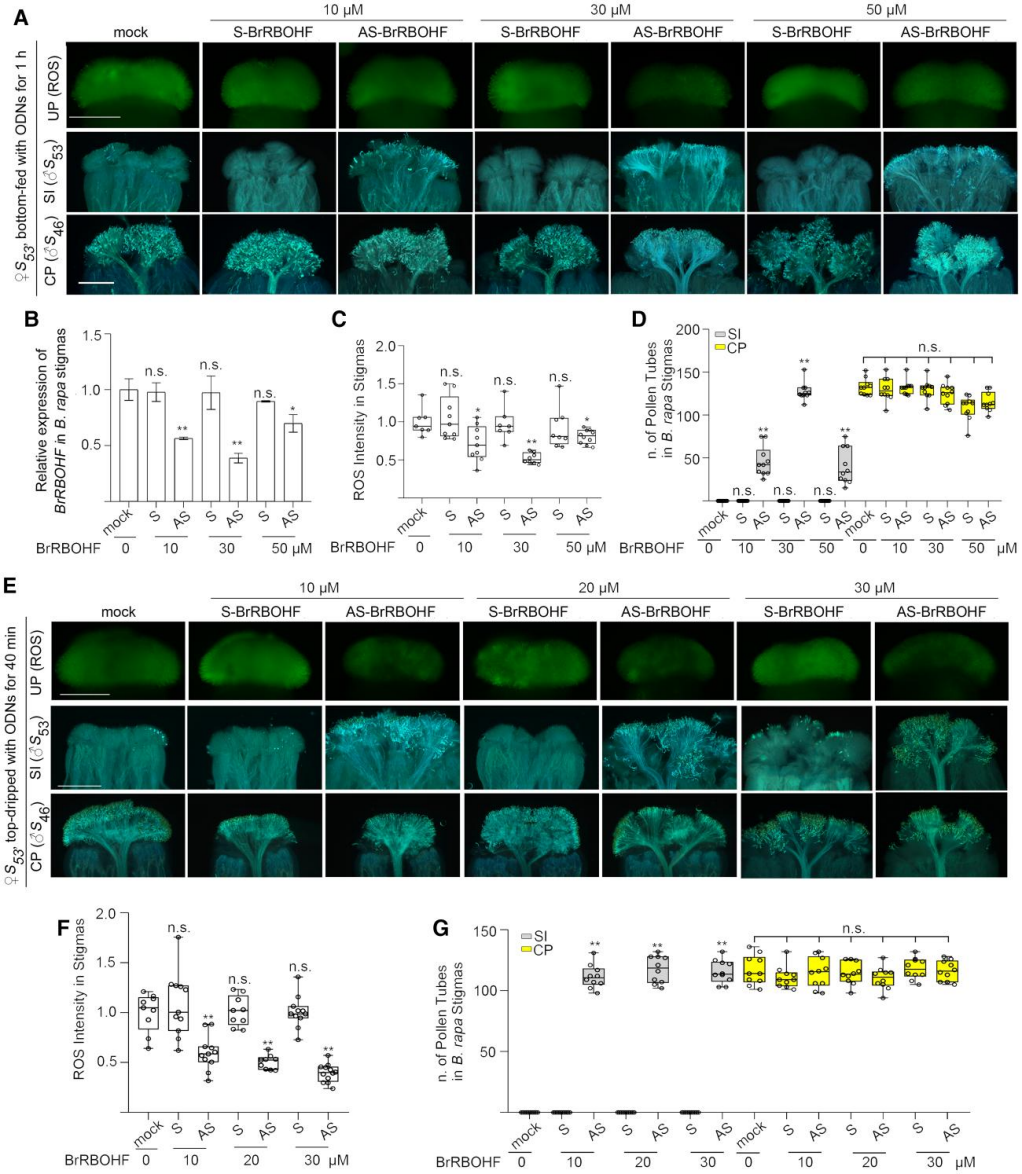
The effect of different concentrations of ODNs targeting BrRBOHF on stigmatic ROS accumulation and self- or cross-pollination in *B. rapa* with bottom-feeding and top-dripping methods. **A)** ROS accumulation and the growth of self- or cross-pollen tubes in stigmas treated with different concentrations of S- or AS-BrRBOHF by the bottom-feeding method. **B)** An RT-qPCR analysis of *BrRBOHF* observed in **A**. Bar graphs: mean value, ±Sd for gene expression data of *BrRBOHF*. The dots indicate each data point. The asterisks in the data indicate a significant difference (2-tailed *t*-test; *P* < 0.05; **P < 0.01); n.s. indicates no significant difference (compared with the mock data on the far left). Each experiment was repeated at least 3 times. **C)** A quantification of ROS signals observed in **A**. **D)** The numbers of self- or cross-pollen tubes observed in **A**. **E)** ROS accumulation and the growth of self- or cross-pollen tubes in stigmas treated with different concentrations of S- or AS-BrRBOHF with the top-dripping method. **F)** A quantification of ROS signals observed in **E**. **G)** The numbers of self- or cross-pollen tubes observed in **E**. Boxplots: center line, median; box limits, upper and lower quartiles; whiskers, highest and lowest data points; dots, individual data points. The asterisks in the data indicate a significant difference (2-tailed *t*-test; **P < 0.01); n.s. indicates no significant difference (compared with the mock data on the far left). Each experiment was repeated at least 3 times. Scale bar = 500 *µ*m.

We first determined the optimum concentration of AS-BrRBOHF in suppressing SI. *B. rapa* stigmas were excised at 1 mm from papilla cells and treated with 10, 30, and 50 *μ*m of AS-BrRBOHF in the bottom-feeding method for 1 h. All 3 concentrations of AS-BrRBOHF significantly suppressed the expression of *BrRBOHF* and reduced ROS levels in *B. rapa* stigmas (Fig. 5, A to C). All 3 concentrations of AS-BrRBOHF were effective in breaking down SI, with stigmas treated with 30 *μ*m AS-BrRBOHF having 3-fold more self-pollen tubes than stigmas treated with 10 and 50 *μ*m (Fig. 5D). In addition, self-pollen tubes in stigma treated with 30 *μ*m of AS-BrRBOHF were also comparable with the numbers of cross-pollen tubes in treated and untreated stigmas (Fig. 5D), suggesting that 30 *μ*m was the optimum concentration for AS-BrRBOHF treatment. In the top-dripping method, stigmas were treated with 10, 20, and 30 *µ*m AS-BrRBOHF for 40 min (Fig. 5E). Similar to that in the bottom-feeding method, the 3 concentrations of AS-BrRBOHF treatments were all comparably effective in reducing ROS and SI (Fig. 5, F and G). Besides, they were all within the optimum range and were able to effectively breakdown SI without inhibiting pollen growth.

### Determine the optimum treatment time for AS-ODN to break down SI

**Figure 6.**
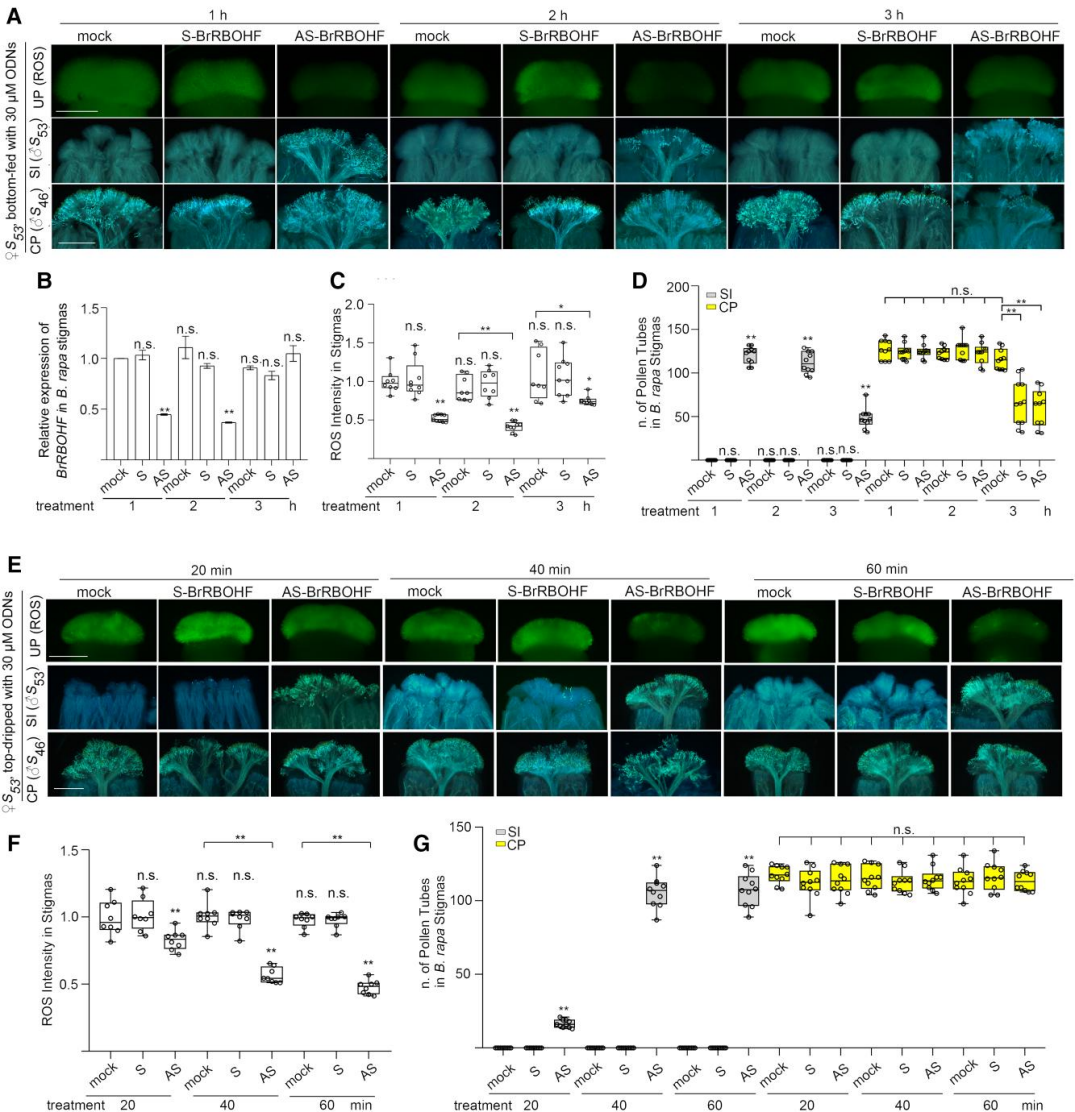
The effect of the treatment time of ODNs on stigmatic ROS accumulation and self-pollination or cross-pollination in *B. rapa* with bottom-feeding and top-dripping methods. **A)** ROS accumulation and the growth of self- or cross-pollen tubes in stigmas treated with S- or AS-BrRBOHF for different durations using the bottom-feeding method. **B)** An RT-qPCR analysis of *BrRBOHF* observed in **A**. Bar graphs: mean value, ±Sd for gene expression data of *BrRBOHF*. The dots indicate each data point. The asterisks in the data indicate a significant difference (2-tailed *t*-test; *P < 0.05; **P < 0.01); n.s. indicates no significant difference (compared with the mock data on the far left). Each experiment was repeated at least 3 times. **C)** A quantification of ROS signals observed in **A**. **D)** The numbers of self- or cross-pollen tubes observed in **A**. **E)** ROS accumulation and the growth of self- or cross-pollen tubes in stigmas treated with ODNs for different durations with the top-dripping method. **F)** A quantification of ROS signals observed in **E**. **G)** The numbers of self- or cross-pollen tubes observed in **E**. Boxplots: center line, median; box limits, upper and lower quartiles; whiskers, highest and lowest data points; dots, individual data points. The asterisks in the data indicate a significant difference (2-tailed *t*-test; *P < 0.05; **P < 0.01); n.s. indicates no significant difference (compared with the mock data on the far left). Each experiment was repeated at least 3 times. Scale bar = 500 *µ*m.

Next, we used 30 *μ*m of AS-BrRBOHF to determine the optimum treatment time in the bottom-feeding methods. *B. rapa* stigmas of 1 mm length were treated with 30 *μ*m AS-BrRBOHF by the bottom-feeding method for 1, 2, and 3 h. The treatment of stigmas with AS-BrRBOHF for 1 and 2 h effectively suppressed the expression of the *BrRBOHF* and reduced stigmatic ROS (Fig. 6, A to C). During both treatment times, the number of self-pollination tubes continued to increase significantly, similar to that of the cross-pollen tubes (Fig. 6D). However, when the treatment was extended to 3 h, the suppression of *BrRBOHF* expression was no longer observed (Fig. 6B). Although the ROS levels were still lower than those of untreated stigmas, they were to a lesser extent compared with stigmas treated for 1 or 2 h (Fig. 6C). The numbers of self-pollen tubes in stigmas treated for 3 h were greater than those in the mock- and S-BrRBOHF-treated controls, but only 1/3 of that in stigmas treated for 1 or 2 h (Fig. 6D). Interestingly, the cross-pollen tube growth was significantly lower in stigmas treated for 3 h with AS-BrRBOHF or S-BrRBOHF compared with all mock treatments and treatments for 1 or 2 h (Fig. 6D), suggesting that the ODNs’ treatment should not exceed 2 h to avoid inhibitory on-pollen growth (Fig. 6, A to D).

In the top-dripping method, treating *B. rapa* stigmas with 30 *μ*m of AS-BrRBOHF for 20, 40, or 60 min, significantly reduced stigmatic ROS levels and promoted the growth of self-pollen tubes in the stigma (Fig. 6, E, F, and G). The number of self-pollen tubes in stigmas treated for 40 and 60 min was ∼5 times the number of self-pollen tubes in stigmas treated for 20 min (Fig. 6G). Furthermore, the number of cross-pollen tubes was comparable in untreated stigmas and stigmas treated for 20, 40, and 60 min (Fig. 6G). From these results, we concluded that 30 *μ*m of AS-BrRBOHF treatment for 1 h effectively suppressed SI in both methods and had no inhibitory effect on pollen growth.

### Spraying ROS scavengers promotes the inbred seed formation of *Brassica* crops

Having established the methodology to modify pollen–stigma interactions with DPI and AS-BrRBOHF in *B. rapa* stigmas, we next explored to what extent reducing ROS of in planta stigmas might promote the formation of self-fertilized seeds. If inbred lines of Brassicaceae vegetables with strong SI are difficult to propagate, they are not suitable to be used as parental lines despite their elite agronomic traits.

**Figure 7.**
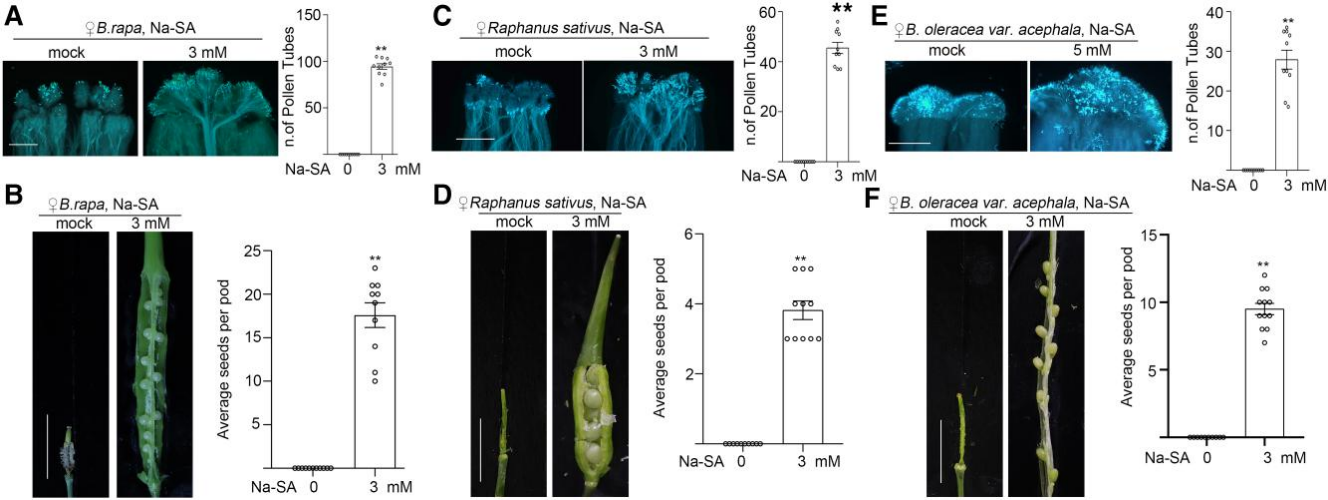
Na-SA to overcome SI in *B. rapa*, *R. sativus*, and *B. oleracea* var. *acephal*a. **A)** The growth of self-pollen tubes in *B. rapa* stigmas treated with Na-SA. **B)** Ovule expansion in the *B. rapa* ovary treated with Na-SA. **C)** The growth of self-pollen tubes in the *R. sativus* stigmas treated with Na-SA. **D)** Ovule expansion in the *R. sativus* ovary treated with Na-SA. **E)** The growth of self-pollen tubes in *B. oleracea* var. *acephala* stigmas treated with Na-SA. **F)** Ovule expansion in the *B. oleracea* var. *acephala* ovary treated with Na-SA. Bar graphs: mean value ± Sem for the pollen tube growth data or numbers of seeds per pod. The dots indicate each data point. The asterisks in the data indicate a significant difference (2-tailed *t*-test; **P < 0.01). Each experiment was repeated at least 3 times. Scale bars in **A**, **C**, and **E**, 500 *µ*m. Scale bars in **B**, **D**, and **F**, 1 cm.

Sodium salicylate (Na-SA) is an effective and inexpensive ROS scavenger. Therefore, we sprayed *B. rapa* pistils with Na-SA to reduce the ROS levels and then pollinated them with self-pollen. By 6 HAP, the self-pollen bundles successfully penetrated the stigmas, and by 12 d after pollination (DAP), enlarged ovules with developing self-fertilized embryos were found in these treated *B. rapa* pistils (Fig. 7, A and B). To validate the broad applicability of this method, we treated the pistils of radish (*Raphanus sativus*) and ornamental kale (*Brassica oleracea* var. *acephala*) with Na-SA in planta (Fig. 7, C to F). These Na-SA-treated pistils exhibited the breakdown of SI and many enlarged ovules with inbred embryos at 12 DAP (Fig. 7, C to F).

## Discussion

Upon pollination, complex interactions between pollen and the stigma initiate to facilitate the growth of compatible pollen and the rejection of SI pollen and interspecies pollen (Huang et al. 2023). Typically, pollen hydration is completed within 20 min, and pollen tube penetration into the papilla cells is completed within 1 h (Zhong et al. 2024). Thus, transient modification in the stigma environment by chemicals may affect pollen recognition, hydration, germination, and penetration.

It is worth noting that cross-pollination is crucial as a control. Due to the inhibitory effect of overdose or prolonged treatment on the growth of self- and cross-pollen, although the chemical treatment successfully breaks down SI, pollen germination and tube elongation on overtreated stigmas may be inhibited. Therefore, cross-pollination must be used as a control in both bottom-feeding and top-dripping treatments to ensure that the treatment is below the upper limit. For example, in *B*. *rapa* stigmas treated with 0, 1, 2, 5, and 10 mm DPI in the bottom-feeding method (Fig. 2, A to C), although DPI reduced stigmatic ROS in a dose-dependent manner, self-pollen could only grow in stigmas with lower DPI concentrations but not in stigmas with high DPI concentrations. This observation would make the role of ROS in SI inconclusive based solely on the growth of self-pollen on stigmas treated with 1, 2, 5, and 10 mm DPI. A good criterion would be that the number of cross-pollen tubes in treated stigmas is comparable with that of untreated stigmas. Below this inhibitory concentration or treatment time, chemical treatments may exhibit a dose-dependent effect to break down SI, i.e. more and more self-pollen tubes would penetrate the stigmas as the concentration or treatment time increases.

For bottom feeding of AS-ODNs, we used 1 mm stigmas rather than 3 mm stigmas in the chemical treatment. Although the time required for chemical transport to the papilla cells in 3 mm long stigmas was much longer than the 1 mm stigmas, stigma length was not a critical factor because the chemicals were generally more stable. The 3 mm long stigmas were easier to handle than the 1 mm ones, but 30 *µ*m AS-BrRBOHF treatment for 1 h failed to suppress the expression of BrRBOHF and failed to reduce ROS in 3 mm stigmas (Fig. 4, A to C). This is because AS-ODNs are easily degraded and move slowly in plant tissues (Mizuta and Higashiyama 2014; Crooke et al. 2017). When handling 1 mm stigmas, the stigmas were simply placed on the surface of PGM medium, rather than inserting the 3 mm stigmas into the medium. Therefore, special care needs to be taken not to drop them or get them wet.

In addition to stigma length, concentration, treatment time, and the sequence design of AS-ODNs should also be considered. When designing the sequences of AS-BrRBOHF, we took into account the high similarity between the sequences of other members of the BrRBOHs family and BrRBOHF. In order to meet the above conditions, we compared the sequences of the BrRBOH family members and designed the conservative region and specific region of BrRBOHF, respectively. It was found that the effect of oligonucleotide treatment in the conserved region was better (Zhang et al. 2021).

## Conclusion

This study elaborated the factors that affect the effectiveness of both bottom-feeding and top-dripping methods, including concentration, duration, and stigma length. Combined with chemical treatment and AS-ODN treatment, this approach is very effective for identifying the signal pathway and the functional genes in pollen–stigma interactions. In addition, the top-dripping method or the spraying method is very useful for breaking down SI and promoting the inbred seed formation. The methods presented in this study provide a convenient approach for further investigating the mechanisms underlying SI and other pollen–stigma interactions.

## Materials and methods

### Plant materials

Two *B. rapa* lines, 14CR and ZY15, were used in this study. The 14CR line was derived from a double-haploid population carrying the *S_46_* haplotype, while ZY15 is a multi-generational inbred line with the *S_53_* haplotype (Zhang et al. 2021). Both varieties exhibit SI in self-pollination, but they are compatible with cross-pollination. During the planting process, fully developed seeds were carefully chosen and then soaked at 50 ℃ for 2 h. Subsequently, the seeds were sown and germinated at room temperature. After these seedlings grew to the 3 to 4 true leaf stage, they were vernalized in an incubator at 4 ℃ for 1 mo. Healthy seedlings were selected for further growth in the greenhouse.

### ODN design and application in stigmas

The ODNs used in this paper were synthesized by the Beijing Institute of Genomics. Sfold (https://sfold.wadsworth.org/cgi-bin/soligo.pl) and BLAST program (https://blast.ncbi.nlm.nih.gov/Blast.cgi) were used to predict the secondary structure and sequence features of ODNs. The sequence of S-BrRBOHF is 5′–ACCAGCACAAGACTATAGAA-3′; and the sequence of AS-BrRBOHF is 5′–TTCTATAGTCTTGTGCTGGT-3′.

The 5′-ends of ODNs were modified with phosphonothioate to prevent degradation and maintain their stabilities. For the bottom-feeding method, PGM including S- or AS-ODN was added to the PCR lids to form a solid agar medium. For the top-dripping method, the stigmas were inserted directly into the solid agar medium (without S- or AS-ODN), and 1 *µ*L solution including S- or AS-ODN was added to the stigma with a pipette. The stigmas were placed in the medium for different periods of treatment before being pollinated and observed under a microscope.

### RNA extraction and RT-qPCR detection

RNA was extracted using the RNAprep Pure Micro Kit (Invitrogen, 12183016). In short, 20 stigmas with a total mass of ∼0.05 g were placed in 500 *µ*L of cracking buffer and mashed with an electric mill. Then, equal volume of 70% ethanol (v/v) was added, and the mix was transferred to the PureLink microkit column and collection tube and centrifuged at 13,400×*g* for 1 min at 4 °C. The pellet was washed twice, followed by a DNase digest step to eliminate DNA contamination. The total RNA was eluted with RNase-free ddH_2_O. The RNA was examined by 1% agarose gel electrophoresis and quantified by Nanodrop 2000C (Thermo Scientific) spectrophotometer.

For the RT-qPCR assay, a 20 *µ*L reaction system [2×ChamQ SYBR qPCR Master Mix (Vazyme) 10 *µ*L, cDNA 0.4 *µ*L, Gene-specific primer 0.4 *µ*L + 0.4 *µ*L, RNase-free ddH_2_O 8.8 *µ*L] was prepared and the qTOWER qPCR machine (Analytikjena, Germany) was used for PCR (95 °C for 30 s, 95 °C for 10 s, and 60 °C for 22 s, with a total of 40 cycles). The experimental data were analyzed by the 2^−ΔΔ^Ct method.

### In vitro treatment of stigmas

Stigmas from flowers that opened on the day of the experiment were collected by carefully cutting them to different lengths below the stigmas using a sharp blade (Huang et al. 2020; Su et al. 2020; Zhang et al. 2021). The stigmas were then inserted into solid PGM medium [5 mm CaCl_2_ (Sigma-Aldrich, 43088), 5 mm KCl (KaiTong, GB/T 1272-200), 0.01% H_3_BO_3_ (Sigma-Aldrich, 10043-35-3), 1 mm MgSO_4_·7H_2_O (Sigma-Aldrich, 10034-99-8), 10% sucrose, and 0.8% agarose, pH 7.5]. For the bottom-feeding method, the medium contained either chemicals or food compound colorant (FLEUR COULEUR, bar code 7971792915206) and was placed in an incubator at a temperature of 22.5 °C and a humidity level of 45% for different durations. At the end of treatment, a portion of the stigmas were stained with H_2_DCFDA (Med Chem Express, HY-D0940), and another portion of stigmas were transferred to the chemical-free medium for self- or cross-pollination for 6 h. The mock medium in the bottom-feeding method has the same amount of the corresponding solvent that did not contain 0.0125% Tween 20. For the top-dripping method, the stigmas were inserted into a solid PGM medium, and a pipette was used to add 1 *µ*L of a chemical solution or food compound colorant containing 0.0125% Tween 20. The mockup in the top-dripping method has the same amount of corresponding solvent containing 0.0125% Tween 20. The stigmas were again placed in an incubator at a temperature of 22.5 ℃ and a humidity level of 45% for different durations. Following this some of the stigmas were stained with H_2_DCFDA, while others were self- or cross-pollinated and cultured for 3 h to observe the number of pollen tubes.

### Aniline blue staining

The stigmas were immersed in Carnoy's Fluid (acetic acid:ethanol = 1:3) for decolorization for a duration of 2 h. Subsequently, they were incubated in 10 m NaOH at the temperature of 42 °C in a water bath for 30 min, following this, the stigmas were stained in a solution of 1% aniline blue (Sigma-Aldrich, 415049) for a period of 1 h (Yan et al. 2023). In order to facilitate the quantification of pollen tubes, we pressed the coverslip further to spread out pollen tubes for easy counting after taking a clear picture of the whole stigma. The observation of pollen tubes was carried out using a Nikon Eclipse Ni microscope equipped with a DS-Ri2 digital camera.

### The observation of ROS staining

The treated stigmas were soaked in a solution of MES-KCl buffer [10 mm MES (Med Chem Express, 4432-31-9), 5 *µ*m KCl, 50 *µ*m CaCl_2_, pH 6.15 adjusted with KOH] for ∼10 min, ensuring that the stigmas were fully submerged in the staining solution. The buffer was then carefully removed by gently aspirating it using a syringe without touching the stigmas. Subsequently, the prepared H_2_DCFDA staining solution (the stock solution of H_2_DCFDA was diluted to a 50 *µ*m working solution with the MES-KCl buffer) was added to stain the stigmas for a 2 h staining period. After staining, the stigmas were washed 3 times with the MES-KCl buffer, with each wash lasting 15 min. The ROS staining was observed using a DS-Ri2 digital camera of a Nikon Eclipse Ni microscope, while eGFP epifluorescence (Ex470-440, DM4951p, BA525/550) was used to observe H_2_DCFDA. The exposure time for imaging was typically set between 800 and 900 ms.

### Quantification and statistical analyses

Boxplots were used to represent the average values of stigmatic ROS, the numbers of pollen tubes, or the average length of pollen tubes. Dots within the boxplots were generated using Prism and represented individual data points. Bar graphs without dots were generated in Excel. Furthermore, ±Sd for all stigmatic ROS data and ±Sem for the numbers or pollen-tube length data were also generated. The asterisks above the box plots indicate significant differences compared with the control data on the far left (2-tailed *t*-test, *P < 0.05, **P < 0.01) and n.s. indicates no significant difference compared with the data bar on the far left. The asterisks or n.s. above the brackets above the box plots indicate the results of comparisons with the data on the far left within the brackets. The experiments were repeated at least 3 times, yielding consistent results. Detailed information of the statistical analysis used in this study is presented in the legend of Fig. 2 and briefly summarized in subsequent figure legends.

### Accession numbers

Sequence data from this article can be found in the GenBank/EMBL data libraries under BrRBOHF accession number Bra027764.

## Supplementary Material

kiae445_Supplementary_Data

## Data Availability

The data underlying this article are available in the article and in its online supplementary material.

## Supplementary data

The following materials are available in the online version of this article.

**Supplementary Figure S1.** Pollen tube growth of stigma in planta, bottom-feeding method, and top-dripping method.

**Supplementary Figure S2.** The absorption of food dye by stigmas in the bottom-feeding and top-dripping methods.

## Dive Curated Terms

The following phenotypic, genotypic, and functional terms are of significance to the work described in this paper:

CALS5 Gramene: AT2G13680
CALS5 Araport: AT2G13680

ROP1 Gramene: AT3G51300
ROP1 Araport: AT3G51300

ANX1 Gramene: AT3G04690
ANX1 Araport: AT3G04690

ANX2 Gramene: AT5G28680
ANX2 Araport: AT5G28680
