# Supplementary material for: Nontransformation methods for studying signaling pathways and genes involved in Brassica rapa pollen–stigma interactions
Source: Plant Physiol. 2024 Aug 30;196(3):1802–12. doi: 10.1093/plphys/kiae445 (PMC11531837; doi:10.1093/plphys/kiae445)
Supplement: kiae445_Supplementary_Data [file kiae445_supplementary_data.pdf]

Supplementary Data

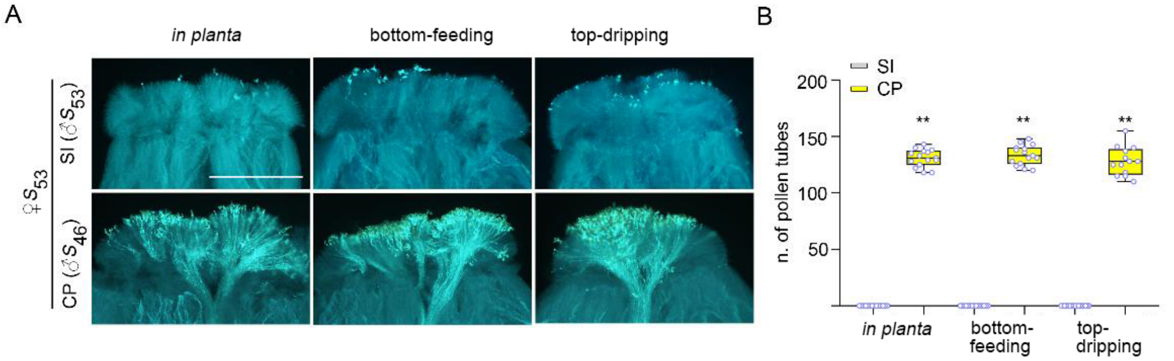

Supplementary Figure S1. Pollen tube growth of stigma in planta, bottom-feeding, and top-dripping methods. A) Aniline blue staining and B) quantification of pollen tubes on stigmas in planta, bottom-feeding, and top-dripping methods. Boxplots: center line, median; box limits, upper and lower quartiles; whiskers, highest and lowest data points; dots, individual data points. Asterisks in the data indicate a significant difference (two-tailed T-test; *p < 0.05; **p < 0.01); n.s. indicates no significant difference (compared to the mock data on the far left). Each experiment was repeated at least three times. Scale bar=500 μm.

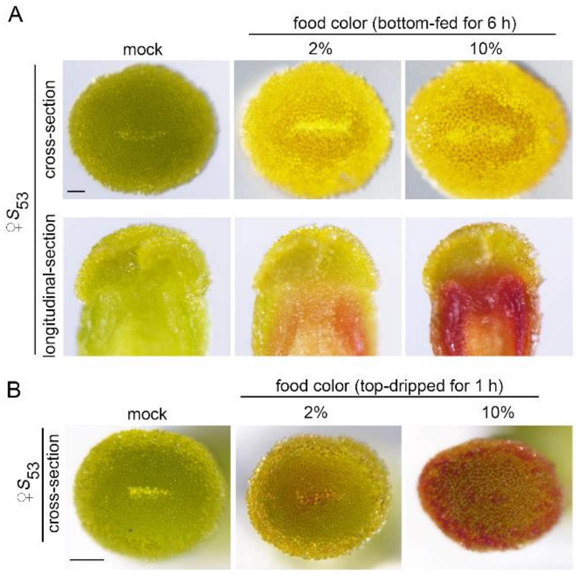

Supplementary Figure S2. The absorption of food dye by stigmas in the bottom -feeding and the top-dripping methods. A) Cross- and longitudinal-sections of stigmas in the bottom-feeding method with food dye. Stigmas treated for 6 h. B) Cross-sections of stigmas in the top-dripping method with food dye. Stigmas were treated for 1 h. Scale bar=100 μm.
